# Contribution of Interferon gamma release assays testing to the diagnosis of latent tuberculosis infection in HIV-infected patients: A comparison of QuantiFERON-TB Gold In Tube, T-SPOT.TB and tuberculin skin test

**DOI:** 10.1186/1471-2334-12-169

**Published:** 2012-07-31

**Authors:** José M Ramos, Catalina Robledano, Mar Masiá, Sofia Belda, Sergio Padilla, Juan C Rodríguez, Félix Gutierrez

**Affiliations:** 1 Infectious Diseases Unit, Hospital General Universitario de Elche, Camino de la Almazara, 12, 03203, Elche, Alicante, Spain; 2 Section of Microbiology, Hospital General Universitario de Elche, Camino de la Almazara, 12, 03203, Elche, Alicante, Spain; 3 Department of Clinical Medicine, University Miguel Hernández, Ctra, Valencia s/n, 03550, San Juan de Alicante, Alicante, Spain

## Abstract

**Background:**

Diagnosis and treatment of latent tuberculosis infection (LTBI) is the most effective strategy to control tuberculosis (TB) among patients with HIV infection. The tuberculin skin test (TST) was the only available method to identify LTBI. The aim of the present work was to evaluate the usefulness of the interferon-gamma release assays (IGRAs): QuantiFERON-tuberculosis (TB) Gold-In-Tube test (QFG) and T-SPOT.TB for the diagnosis of LTBI in a diverse cohort of HIV-infected patients.

**Methods:**

A prospective study was carried out in consecutive patients cared for in a single institution in Spain from January 2009 to October 2010. IGRAs and TST were performed simultaneously. TST induration ≥ 5 mm was considered positive.

**Results:**

QFG, T-SPOT.TB and TST were performed in 373 subjects. Median CD4 cell count was 470/μl with a median nadir of 150/μl. TST, QFG and T-SPOT.TB were positive in 13.3%, 7.5% and 18.5% cases respectively. Among 277 patients with neither past or current TB nor previous treatment for LTBI and who had TST results, a positive TST result was obtained in 20 (7.2%) cases. When adding QFG results to TST, there were a total of 26 (8.6%) diagnoses of LTBI. When the results of both IGRAs were added, the number of diagnoses increased to 54 (17.9%) (incremental difference: 10.7% [95% confidence interval [CI]:5.3-16.2%] [p < 0.001]), and when both IGRAs were added, the number of diagnoses reached 56 (18.5%) (incremental difference: 11.3% [95% CI:5.7%–16.9%] [p < 0.001]). Patients with a CD4 cell count greater than 500 cells/μl and prior stay in prison were more likely to have a diagnosis of LTBI by TST and/or QFG and/or T-SPOT.TB (adjusted odds ratio [aOR]: 3.8; 95% CI, 1.4 – 9.9; and aOR: 3.3; 95% CI, 1.3 – 8.3, respectively).

**Conclusions:**

IGRAs were more sensitive than TST for diagnosis of *M. tuberculosis* infection in HIV-infected patients. Dual sequential testing with TST and IGRAs may be the optimal approach for LTBI screening in this population.

## Background

Diagnosis and treatment of latent tuberculosis infection (LTBI) is the most effective strategy to control tuberculosis (TB) among patients with HIV infection [1,2]. The tuberculin skin test (TST), the only available method to identify LTBI for more than a century, is known to have major constraints [3], including a reduced sensitivity in HIV-infected patients as compared with the general population, particularly in those with low CD4 cell counts [4].

The development of *in vitro* blood tests to evaluate cell-mediated immune response against *Mycobacterium tuberculosis* (*M. tuberculosis*) has been a major advance for the diagnosis of LTBI. The interferon-gamma release assays (IGRAs) measure T-cell release of interferon-γ (IFN-γ) following stimulation by antigens that are unique to *M. tuberculosis* including early-secreted antigenic target 6 (ESAT-6) and culture filtrate protein 10 (CFP-10), which are encoded by genes located within the region of difference 1 (RD1) segment of the *M. tuberculosis* genome [5]. These antigens are more specific for *M. tuberculosis* than those in the purified protein derivate (PPD) used in the TST because they are not shared with any BacilleCalmette-Guérin (BCG) vaccine strains. However, at least 3 species of nontuberculous mycobacteria also have the RD1 antigens as *M. kansasii*, *M. marinum* and *M. szulgai*. QuantiFERON-TB Gold In-Tube test (QFG) (Cellestis, Carnegie, Australia) measures the level of soluble IFN-γ produced in whole blood by enzyme-linked immunosorbent assay (ELISA), and the T-SPOT.TB (Oxford Immunotec, Abingdon, UK) assay detects the number of IFN-γ -producing cells represented as spot-forming units (SFU). The IGRAs present practical and theoretical advantages over TST, and the US Centers for Disease Control (CDC) have released guidelines for using these assays to detect *M. tuberculosis* infection in certain circumstances [6].

Published data on the performance of the IGRAs in patients with HIV infection are limited and discordant [7-10]. Moreover, only a few studies have compared both IGRAs with TST in the diagnosis of LTBI among HIV-infected individuals [11-13]. To determine the real performance of these tests for the diagnosis of LTBI in HIV-infected patients, large studies that include a consecutive series of patients with a broad spectrum of HIV disease should ideally be performed. Given the reduced sensitivity of TST in those patients, evaluating the contribution of IGRAs to conventional TST is critical to decide if these tests should be incorporated into clinical practice in HIV medicine. To our knowledge, there is little information. The aim of the present work was to evaluate the usefulness of the IGRAs in a diverse cohort of HIV-infected patients. In this investigation, patients with different stages of HIV disease were studied to assess the tests, and the characteristics of patients that provided a positive IGRAs result were carefully examined.

## Methods

### Patients and samples

Patients were recruited into the study at the outpatient HIV clinic of a university hospital (Hospital General Universitario de Elche, Alicante, Spain). Eligible patients were all HIV-infected adults (age ≥ 15 years) cared for in the clinic from January 2009 to October 2010. The study was approved by the Ethics Committee of Hospital General Universitario de Elche, and all the patients gave their written informed consent.

Demographical and clinical data including age, sex, nationality, HIV transmission route, years from diagnosis of HIV infection, CDC category, AIDS events, and antiretroviral treatment (ART) received prior to the study were recorded from the patients. Details were taken of previous treatment for positive TST or past or current TB, BCG vaccination status, history of household contact with TB cases, intravenous drug use, and history of previous stay prison or drug rehabilitation unit. Data were also collected about immunologic status (nadir CD4 cell count, CD4 cell count and percentage at the time of evaluation) and plasma HIV-1 RNA viral load. The past TB was referred by the patient or collected from clinical records. Current TB diagnosis was based on the results of clinical and radiologic examination and/or isolation of *M. tuberculosis* from sputum or other specimen (urine, lymph node and cerebrospinal fluid). LTBI was defined by a previous positive TST performed in our clinic or in another clinic.

### Procedures

Blood samples were collected for measurement of the IGRAs tests QFG and T-SPOT.TB and a conventional TST was performed simultaneously in a blinded fashion. Study participants were injected with 0.1 ml of tuberculin (2 tuberculin units of PPD) (Tuberculina PPD; Evans 2UT, UCB Pharma, S.A. Madrid, Spain) in accordance with the American Thoracic Society guidelines [14]. The skin induration was measured with a rule at 48-72 hours after the inoculation. The size of the induration equal or higher than 5 mm was considered positive. The previous BCG vaccination did not change the size limits for the tuberculin reaction. The result was considered to be invalid or not read, if the patient did no come to measure the induration and was impossible to contact him/her. All patients with a positive tuberculin test who had not been previously treated, received chemoprophylaxis with isoniazid for 6-9 months. Patients with a positive IGRA plus a positive TST also received isoniazid chemoprophylaxis. Their doctors in charge, who decided to start chemoprophylaxis or not according to additional accompanying factors individually managed patients with a positive IGRA, but negative TST.

For QFG, whole blood was collected from each patient and inoculated in three heparinized tubes of 1 ml each: one containing TB antigens (ESAT-6, CFP-10, and TB7.7), a positive control tube containing phytohemagglutinin, and a null control. Blood samples were incubated for 16-20 h at 37°C. Plasma samples were then harvested for IFN-γ quantification by a single-step sandwich-type ELISA. The test was performed according to manufacturer’s instructions (Cellestis, Carnegie, Australia) [15]. Optical densities were interpreted using specific software provided by the manufacturer. The result was considered to be positive if the IFN-γ level after stimulation with TB antigens minus negative control was ≥ 0.35 IU/ml and ≥ 25% of the negative control. The test was considered negative if the IFN-γ level was < 0.35 IU/ml (after subtraction of the negative control). The test result was considered to be indeterminate if (1) the negative control was ≥ 8.0 IU/ml or (2) the positive control was < 0.5 IU/ml.

The T-SPOT.TB test was performed according to the recommendations of the manufacturer (Oxford Immunotec, Abingdon, UK) [16]. We use T-Cell Xtend (Oxford Immunotec, Abingdon, UK) reagent that is added to blood samples in the laboratory immediately before running the T-SPOT.TB assay. It allows blood samples to be processed up to 32 hours after venopuncture without affecting the accuracy of the test. Concisely, peripheral blood mononuclear cells were spared by centrifugation from an 8 ml heparinized blood sample and then placed into four wells (2.5x10^5^ cells per well). The wells were stimulated with 50 μL each of phytohemagglutinin (positive control), ESAT-6, CFP-10, and AIM® V medium (Invitrogen. USA) (negative control). The wells were incubated for 16-20 h at 37°C in 5% CO2, washed, and developed with a conjugate against the antibody used and an enzyme substrate. The SFUs were counted with an USB Microscope (MicroCapture) [17], by one observer in case of doubt by two observers. The results of T-SPOT-TB were interpreted according to the following criteria:

(1) Manufacturer’s criteria [16]. A result was considered to be positive if the number of SFUs was > 5 (after subtraction of the SFUs of the negative control). If the negative control well was between 6 and 9 SFUs, the result was considered positive if the number of SFUs in the antigen well was > 2 x SFUs negative-control. The test result was considered to be negative if the above criteria were not met and the positive control was valid. The result was considered to be invalid if the SFUs of the negative control was >10 or if the SFUs of the positive control well was < 20 SFUs.

(2) Food and Drugs Agency (FDA) of the US criteria [18]. These interpretation criteria included a borderline interpretation. A result was considered to be positive if the number of SFUs was ≥ 8 (after subtraction of the SFUs of negative control). The result was considered borderline if SFUs was equal to 5, 6 or 7. The result was considered negative if the number of SFUs was ≤ 4. The result was considered to be invalid if the number of SFUs of negative control was >10 or if < 20 SFUs in the positive control well.

### Statistical analysis

Data were analyzed using the SPSS software package version 12.0 (SPSS Inc., Chicago, Illinois, USA). Concordance between dichotomized TST, QFG and T-SPOT.TB was assessed by kappa (κ) coefficient. Strength of agreement was considered ‘poor’ for κ ≤ 0.20, ‘fair’ for 0.20 < κ ≤ 0.40, ‘moderate’ for 0.40 < κ ≤ 0.60, ‘substantial’ for 0.60 < κ ≤ 0.80, and ‘optimal’ for 0.80 < κ ≤ 1.00 [19]. For the analysis of agreement indeterminate and invalid tests results were excluded.

Fisher’s exact test, Chi-square for lineal association, Mc Nemar’s test, and Kruskal-Wallis test were used for comparisons where appropriate. Significant univariate predictors (P < 0.05) were included in a logistic regression model to identify independent predictors measured by odds ratio (adjusted odds ratio, [aOR]) with the 95% confidence interval (CI). We calculate the lower and upper limits of the 95% CI for a proportion and the z-ratio for the significance of the difference between two proportions.

## Results

### Study population

The three tests, QFG, T-SPOT.TB and TST, were performed in 373 subjects: Demographic and clinical characteristics of these patients are summarized in Table 1. The majority of patients were Spanish (92.5%); 187 (50.1%) had history of intravenous drug use, and 144 (38.6%) had a prior AIDS-defining event. Most of them (74.5%) were on ART.

**Table 1:** Baseline demographic and clinic characteristics of 373 HIV-infected patients undergoing tuberculin skin test, QuantiFERON-TB Gold and T-SPOT-TB testing, stratified according to past or current tuberculosis (TB) and previous positive tuberculin skin test (TST)

	**All patients (n = 373)**	**Patients with past or current TB (n = 56)**	**Patients with positive TST treated (n = 15)**	**Patients with no past or current TB nor positive TST (n = 302)**	P value*
**Demography**					
Median age, years (range)	44 (15-85)	45 (21-74)	42 (27-50)	44 (15-85)	NS
Sex, male	287 (76.9)	44 (78.6)	14 (93.3)	229 (75.8)	NS
Origin from a country with high prevalence of TB	28 (7.5)	7 (12.5)	1 (6.7)	20 (6.7)	NS
**HIV-related factors**					
Prior AIDS defining illness	144 (38.6)	50 (89.3)	4 (26.7)	92 (30.5)	<0.001
Median CD4 cell count, cells/μl (range)	470 (10-1760)	350	442 (120-1200)	500 (10-1760)	0.02
		(10-1550)			
CD4 cell count < 200/μl	62 (16.6)	20 (35.7)	3 (20)	39 (12.9)	<0.001
CD4 cell count < 350/μl	135 (36.2)	28 (50)	9 (60)	101 (33.4)	0.03
CD4 cell count < 500/μl	198 (53.1)	35 (62.5)	10 (66.7)	153 (50.7)	0.07
Median nadir CD4 cell count, cells/μl (range)	150 (5-1650)	70 (5-1220)	240 (42-500)	180 (5-1650)	<0.001
Median HIV-1 ARN viral load, copies/ml (range)	<50 (0-1 x 10^6^)	115 (0-1 x 10^6^)	<50 (0-85486)	<50 (0-1 x 10^6^)	NS
HIV-1 RNA viral load < 50 copies/ml	218 (58.4)	35 (49.3)	11 (74)	183 (60.6)	NS
ART-naïve	95 (25.5)	24 (42.8)	13 (86.7)	77 (25.5)	NS
Median time (years) since 1^st^ HIV-positive test (range)	10 (0-27)	12 (0.1-22)	12 (3-24)	10 (0.1-27)	0.001
BCG vaccine	58 (15.8)	13 (23.2)	5 (33.3)	40 (13.9)	0.02
**Risk factors for TB infection**					
Contact with patients with TB	144 (38.7)	40 (71.4)	11 (17.3)	93 (30.9)	<0.001
History of injection drug use	187 (50.1)	35 (62.5)	14 (93.3)	138 (45.7)	0.001
History of prior prison stay	102 (27.3)	29 (51.8)	13 (86.7)	60 (19.9)	<0.001
Lived in a shelter or homeless	97 (26.0)	22 (39.2)	6 (40)	66 (21.9)	<0.001
Diabetes mellitus	16 (4.3)	1 (1.1)	1 (6.7)	14 (4.6)	NS
Others					
Hepatitis C virus co-infection	167 (45.1)	33 (58.9)	14 (93.3)	120 (40.0)	<0.001

NOTE. Data are no (%) of patients unless otherwise indicated; ART, antiretroviral therapy; BCG, Bacille Calmette-Guérin; * p-value compared the patients with past or current TB and previous positive TST with patients with no past or current TB nor positive TST.

The median HIV-1 RNA viral load was <50 copies/ml (range, <50 – 1 x 10^6^); 218 (58.4%) patients had HIV-1 RNA viral load < 50 copies/ml. The median CD4 cell count was 470 cells/μl (range, 10–1760/μl); 62 (16.6%) individuals had CD4 cell count < 200 cells/μl. There were 71 patients with either: a history of previously active TB (n = 50), a history of previous treatment for LTBI (n = 15), and current active TB (n = 6). Table 1 shows the differences between those patients without and with past (n = 50) TB or current TB (n = 6) or previous positive TST treated (n = 15).

### Diagnostic tests

**Figure 1:**
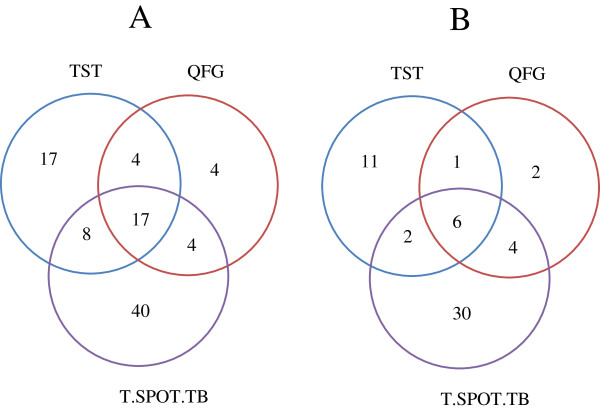
Venn diagram illustrating how often the tuberculin skin test (TST), QuantiFERON-TB Gold (QFG), and T-SPOT.TB tests coincide in terms of positives as defined by the manufacturers in 372 HIV-infected patients (A) and in 302 HIV-infected patients with no past or current TB nor treated for of latent tuberculosis infection (B).

A TST result was not available in 28 (7.5%) of 373 patients, the reason being in all the cases that the patient did not come to measure the induration, and it was impossible to contact him/her. In 13.3% (46/345) patients the TST was positive (induration of at least 5 mm). QFG was positive in 7.5% (28/373) patients. T-SPOT.TB was positive in 18.5% (69/373) patients according to manufacturer’s criteria. When T-SPOT.TB results were categorized according to FDA’s criteria, 13.4% (50/373) patients gave a positive result, and 7.2% (27/373) a borderline result. In the Figure 1 shows a Venn diagram where the various tests coincide in terms of positives as defined by the manufacturers. QFG and T-SPOT.TB were more often positive in patients with past or current TB or previous treatment for positive TST and in patients with contact with TB patients as summarized in Table 2.

**Table 2:** Comparison of tuberculin skin test (TST), QuantiFERON-TB Gold (QFG), and T-SPOT.TB in all the patients, in patients with past or current tuberculosis (TB) or previous positive tuberculin skin test (TST) and in patients with no past or current TB nor treatment for LTBI with and without TB contact

**Test**	**Patients**	**Results of Test**			
		**Positive**	**Borderline**	**Negative**	**Indeterminate or invalid**
TST	All patients (n = 373)	46 (12.3)	-	299 (80.2)	28 (7.5)
	Patients with past or current TB or positive TST (n = 71)	26 (36.6)	-	42 (59.2)	3 (4.2)
	Patients with no past or current TB nor positive TST without TB contact (n = 209)	7 (3.3)		188 (90.0)	14 (6.7)
	Patients with no past or current TB nor positive TST with TB contact (n = 93)	13 (14.0)	-	69 (74.2)	11 (11.8)
QFG	All patients (n = 373)	28 (7.5)	-	335 (89.8)	10 (2.7)
	Patients with past or current TB or positive TST (n = 71)	15 (21.1)	-	56 (78.9)	0 (0)
	Patients with no past or current TB nor positive TST without TB contact (n = 209)	6 (2.9)		198 (94.7)	5 (2.4)
	Patients with no past or current TB nor positive TST with TB contact (n = 93)	7 (7.5)	-	81 (87.1)	5 (2.4)
T-SPOT.TB (Mc)	All patients (n = 373)	69 (18.5)	-	278 (74.5)	26 (7.0)
	Patients with past or current TB or positive TST (n = 71)	27 (38.0)	-	40 (56.3)	4 (5.6)
	Patients with no past or current TB nor positive TST with TB contact (n = 209)	28 (13.4)		166 (74.4)	15 (7.2)
	Patients with no past or current TB nor positive TST with TB contact (n = 93)	14 (15.1)	-	72 (77.4)	7 (7.5)
T-SPOT.TB (FDAc)	All patients (n = 373)	50 (13.4)	27 (7.2)	270 (72.4)	26 (7.0)
	Patients with past or current TB or positive TST (n = 71)	22 (31.0)	7 (9.9)	38 (53.5)	4 (5.6)
	Patients with no past or current TB nor positive TST without TB contact (n = 209)	20 (9.6)	12 (5.7)	162 (77.5)	15 (7.2)
	Patients with no past or current TB nor positive TST with TB contact (n = 93)	8 (8.6)	8 (8.6)	70 (72.9)	7 (7.5)

NOTE. Data are no (%) of patients unless otherwise indicated.

Mc: Manufacturer’s interpretation criteria; FDAc: Food and Drug Agency’s interpretation criteria.

TST, QFG and T-SPOT.TB were positive in 13.3%, 7.5% and 18.5% cases; respectively. The number of indeterminate or invalid results was lower for QFG than for T-SPOT.TB (2.7% versus 7.2%; p = 0.002). An indeterminate QFG result was statistically associated with CD4 cell count < 200/μl (8.1% versus 1.6%; p < 0.001). No association of low CD4 cell count with indeterminate T-SPOT.TB results was found.

Table 3 illustrates the concordance between TST, QFG and T-SPOT.TB results stratified according to medical history of past or current TB or previous treatment for positive TST. Overall, agreement between TST and the IGRAs was moderate or poor. It was higher in patients with past or current TB or previous treatment for LTBI, in whom the tests were more likely to be positive. Of note, there was poor concordance between the two IGRAs (κ = 0.351), particularly when using the manufacturer’s interpretation criteria for T-SPOT.TB.

**Table 3:** Concordance between tuberculin skin test (TST), QuantiFERON-TB Gold (QFG) and T-SPOT.TB in all the patients, in patients with past or current tuberculosis (TB) or previous treatment for latent TB infection (LTBI), and patients with no past or current TB nor treatment for LTBI

	**All patients**	**Patients with past or current TB or treatment for LTBI**	**Patients with no past or current TB nor treatment for LTBI**
QFG *vs TST*	0.548	0.524	0.437
	(0.417-0.621)	(0.359-0.615)	(0.233-0.663)
T.SPOT.TB (Mc)* vs TST*	0.397	0.415	0.238
	(0.268-0.515)	(0.233-0.663)	(0.092-0.392)
T.SPOT.TB (FDAc)** vs TST*	0.483	0.493	0.359
	(0.339-0.609)	(0.249-0.685)	(0.176-0.541)
T.SPOT.TB (FDAc)*** vs TST*	0.392	0.482	0.237
	(0.269-0.500)	(0.243-0.665)	(0.100-0.372)
T.SPOT.TB (FDAc)**** vs TST*	0.455	0.430	0.350
	(0.315-0.583)	(0.186-0.622)	(0.171-0.533)
T.SPOT.TB (Mc)* vs QFG*	0.365	0.331	0.324
	(0.255-0.437)	(0.110-0.483)	(0.196-0.381)
T.SPOT.TB (FDAc)** vs QFG*	0.478	0.456	0.426
	(0.348-0.554)	(0.213-0.601)	(0.259-0.504)
T.SPOT.TB (FDAc)*** vs QFG*	0.351	0.355	0.289
	(0.250-0.411)	(0.143-0.477)	(0.171-0.341)
T.SPOT.TB (FDAc)**** vs QFG*	0.464	0.447	0.414
	(0.336-0.560)	(0.208-0.611)	(0.245-0.515)

NOTE. Data are kappa (κ) coefficient and lower and upper limits of the 95% confidence interval.

Mc: Manufacturer’s interpretation criteria; FDAc: Food and Drug Agency’s interpretation criteria.

*Excluding indeterminate results; ** excluding invalid and borderline results; *** excluding invalid results and borderline results considered as positive; **** excluding invalid results and borderline results considered as negative.

### Contribution of IGRAs to the diagnosis of LTBI

**Figure 2:**
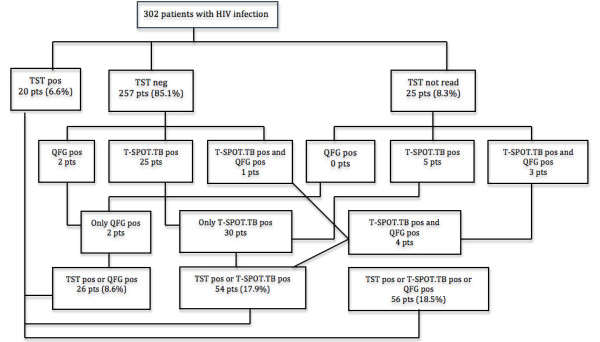
Flow chart illustrating early and final diagnosis of latent tuberculosis (TB) infection (LTBI) in 302 HIV-infected patients with no past or current TB nor treated for LTBI when adding QuantiFERON-TB Gold (QFG) and T-SPOT.TB to tuberculin skin test (TST).

To assess the contribution of IGRAs to the diagnosis of LTBI, only 302 patients with no past or current TB nor previous treatment for positive TST were analyzed. A TST result was not available in 25 (8.3%) and a positive result was obtained in 7.2% (20/277). In the Figure 2 shows how often the various tests coincide in terms of positives in these 302 patients. When, we added QFG results to TST, there were a total of 26 (8.6%) diagnoses of LTBI (incremental difference: 1.4% [95% CI: -3.1% – +5.7%] [p = 0.5]). When adding T.SPOT.TB results interpreted according to manufacturer’s criteria, the number of cases of LTBI increased to 54 (17.9%) (incremental difference: 10.7% [95% CI: 5.3%-16.2%] [p < 0.001]), and when both QFG and T-SPOT.TB interpreted according to manufacturer’s criteria were added, the number of diagnosis of LTBI reached 56 (18.5%) (incremental difference: 11.3% [95% CI: 5.7% – 16.9%] [p < 0.001]). When the more restricted T-SPOT.TB FDA´s interpretation criteria were used, the number of diagnosis of LTBI was 40 (13.2%) (incremental difference: 6.0% [95% CI: 0.8% - 8.2%] [p = 0.02]), and after considering both QFG and T-SPOT.TB interpreted according to FDA´s criteria, it was 42 (14.6%) (incremental difference: 6.7% [95% CI: 1.4% – 11.9%] [p = 0.009]).

### Factors associated with LBTI diagnosis by TST, QFG or T-SPOT.TB

Table 4 shows the significantly factors associated with LBTI diagnosed by TST and/or IGRAs. Compared with patients with a TST negative, a LBTI diagnosis by a TST positive (n = 20) was more common in those with a higher median current CD4 (630 [range, 90-1650] versus 480 [10-1760]) (p = 0.02), a longer time from the first HIV-positive test (11.5 [range, 0.1-27] versus 10 [range, 0-25]) (p = 0.04), and in those with history of contact with patients with TB (65% versus 28.5%) (p = 0.002), history of injection drug use (75% versus 43.6%) (p = 0.02), prior prison stay (60% versus 17%) (p < 0.001), those living in a shelter or being homeless (55% versus 19.5%) (p < 0.001), and with hepatitis C virus co-infection (70% versus 37.9%) (p = 0.009). In logistic regression analysis, a history of contact with patients with TB (aOR 4.6; CI 95%: 1.18-18.5) and prior prison stay (aOR 4.4; CI 95%: 1.18 – 18.7) were independently associated with a positive TST.

**Table 4:** Significant variables associated with latent tuberculosis infection (LTBI) stratified by tuberculin skin test (TST) or interferon-gamma release assays in 302 HIV-infected patients with no past or current tuberculosis (TB) nor LTBI

	**TST positive**	**QFG or T-SPOT.TB**	**Either TST, QFG or T-SPOT.TB**	**All tests (TST, QFG and T.SPOT.TB (Mc) negative**
	(n = 20) *	**(Mc) positive**	**(Mc) positive**	
		(n = 45) **	(n = 56) ***	**(n = 246)**
Median CD4 cell/μl count (range)	**630 (90-1650)†**	**580 (10-1740) ‡**	**580 (10-1730) †**	450 (10-1760)
CD4 cell count < 350/μl	4 (20)	**9 (20)¢**	13 (23.2)	88 (35.8)
CD4 cell count < 500/μl	6 (30)	**12 (26.5) ‡**	**18 (32.1) §**	135 (54.9)
Median time (year) from 1^st^ HIV-positive test (range)	**11.5 (0.1-27) ††**	**12 (0.1-25) c**	**12 (0.1-27) ††**	9.5 (0.1-23)
Contact with patients with TB	**13 (65) §**	4 (8.9)	**24 (42.9) ††**	69 (28.2)
History of injection drug use	**15 (75) †**	24 (53.3)	**33 (58.8) ††**	105 (42.7)
History of prior prison stay	**12 (60) ‡**	24 (53.3)	**21 (37.1) §**	39 (17.9)
Lived in a shelter or homeless	**11 (55) ‡**	10 (25.2)	18 (32.1)	48 (19.5)
Hepatitis C virus co-infection	**14 (70) ¢**	20 (44.5)	28 (50.0)	92 (37.7)

NOTE. Data are no (%) of patients unless otherwise indicated.

ART, antiretroviral therapy; BCG, Bacille Calmette-Guérin; QFG, QuantiFERON-TB Gold, Mc: Manufacturer’s interpretation criteria.

The numbers printed in bold letters indicate the value of variables significantly associated with positive test results in univariate analyses: †† p = 0.04; † p = 0.02 § p = 0.002; ¢ p = 0.009; ‡ p < 0.001; * comparing TST positive (n = 20) with TST negative patients (n = 282); ** comparing QFG or T.SPOT.TB positive (n = 45) with QFG or T.SPOT-TB (Mc) negative patients (n = 257); *** comparing either TST, QFG or T-SPOT.TB (Mc) positive (n = 56) with all tests (TST, QFG and T.SPOT.TB-Mc) negative (n = 246).

A statistically significant association was found between a LTBI diagnosis by either TST or QFG or T.SPOT.TB and a higher median current CD4 (580 [range, 10-1730] versus 450 [range, 10-1760] (p = 0.02), a CD4 cell count greater than 500 cell/μl (67.9% versus 45.1%) (p = 0.002), longer time from the first HIV-positive test (12 [range, 0.1-27] versus 9.5 [range, 0.1-23] years) (p = 0.04), history of contact with patients with TB (42.9% versus 28.2%) (p = 0.04), history of injection drug use (58.8% versus 42.7%) (p = 0.04) and prior prison stay (37.1% versus 17.9%) (p = 0.002). In logistic regression analysis, patients with a CD4 cell count greater than 500 cells/μl and prior stay in prison were more likely to have a diagnosis of LTBI (aOR 3.8; CI 95%: 1.4 – 9.9, and aOR 3.3; CI 95%: 1.3 – 8.3, respectively).

## Discussion

To our knowledge this is the largest study to date to evaluate QFG and T-SPOT.TB tests and to define its usefulness, compared with that of TST, in the diagnosis of LTBI in HIV-infected patients in a middle-low TB incidence setting. We found that IGRAs were more sensitive than TST for the diagnosis of *M. tuberculosis* infection. T-SPOT.TB test gave the largest number of positive results using either manufacturer’s criteria or FDA’s criteria. Noteworthy, T-SPOT.TB was positive in many patients with a negative TST result, thus indicating that dual sequential testing with TST and IGRAs may be the optimal approach for LTBI screening in HIV-infected patients. Previous studies had shown that IGRAs have higher specificity than TST in low TB incidence settings [7,9,10,12,13,20], but did not specifically assess the contribution of IGRAs to the diagnosis of LTBI in patients with HIV infection. Therefore, this study expands upon the evaluation of IGRA and offers relevant information on its potential clinical usefulness.

As found in previous studies performed in HIV-infected patients [10,12,13,21], agreement between the IGRA tests and TST was moderate or poor, and indeterminate test results occurred in a significant proportion of the patients. As expected [12,13], a higher rate of indeterminate results was found with the T-SPOT.TB test than with QFG. In agreement with previous studies [12,13,20] indeterminate results of QFG were associated with CD4 count of < 200 cell/μl, while the same did not occur for T-SPOT.TB test. By contrast, indeterminate results for T-SPOT.TB have been related to older age and conditions of transportation [22].

In our study, T-SPOT.TB outperformed QFG. The proportion of individuals testing positive has also been found significantly greater with T-SPOT.TB than with QFG in other studies conducted in asymptomatic HIV-infected patients [11-13,20]. The higher performance of T-SPOT.TB with respect to QFG is in line with previous data indicating that T-SPOT.TB may be less affected by advanced immunosuppression [21,23], and supported by investigations conducted in persons with culture-confirmed active TB [24,25]. In a recent meta-analysis, pooled sensitivity estimates in culture-confirmed active TB were higher for T-SPOT.TB than for QFG [21]. In studies carried out in countries with low prevalence of TB, sensitivity was 94% for T-SPOT.TB [24]. In the study of Sauzullo et al the sensitivity of QFG was 67%, not higher than that of TST in head-to-head comparison [25]. It should be stated, however, that we did not use the most reliable method for counting SFU, the automated ELISPOT plate reader, and therefore misclassification of some of our results can not be ruled out.

Studies evaluating new laboratory techniques used for the diagnosis of LTBI face a major challenge, which is the lack of a satisfactory “gold standard” for assessing both sensitivity and specificity, particularly in HIV-infected patients. The patients with past, current history of TB or LTBI had a higher frequency of positive tests presumably because of a persistent immune response. Those patients were included in the study as “positive controls”, to assess the yield and reliability of the tests in the subset of patients who retained persistent immune response.

Whereas prospective cohort studies have shown that persons with a positive TST result have an increased risk of developing active TB compared with persons with negative TST result [1,26], there is a general agreement that TST has low sensitivity, and it is assumed that TST positive cases represent only a portion of the cases of LTBI. Based on the test-positive rate among patients with culture-confirmed TB, IGRAs are considered to be more sensitive than TST [27]. Indeed, in our cohort, T-SPOT.TB was more frequently positive than TST, particularly in patients with no past or current TB nor treatment for LTBI.

A number of studies have pointed out that IGRAs may also have a higher specificity than TST for the diagnosis of LTBI in low TB incidence settings and correlate better with surrogate markers of *M. tuberculosis* exposure [27,28]. Although data from large prospective cohort studies evaluating the clinical impact and predictive value of IGRA testing are currently lacking, available information suggests that these tests may predict the subsequent development of active TB in certain patient populations even better than TST [29,30]. In a recent study in male patients with silicosis without clinical suspicion of active TB, past history of TB, and treatment for LTBI, T-SPOT.TB outperformed TST in predicting TB disease [29], and a recent meta-analysis that included studies of HIV-infected and HIV–uninfected patients found that IGRAs results are more strongly associated with progression to active TB than TST results [30].

Interestingly, in our study, T-SPOT.TB increased the detection of *M. tuberculosis* infection in patients with negative TST by 7.3% when using the FDA’s interpretation criteria or by 11.9% according to the manufacturer’s interpretation criteria. There is little information on the significance of a positive IGRAs result with negative TST in HIV-infected individuals. Four previous longitudinal studies have evaluated the ability of IGRAs to predict future development of active TB in HIV-infected individuals and all of them reported a higher risk of active TB in individuals with positive IGRAs results [24,31-33]. However, in all of the 3 studies the duration of follow-up was limited and had few incident cases of active TB [21]. Unfortunately, our study design did not allow evaluating the clinical impact of implementing IGRAs in LTBI screening, neither the predictive value for active TB of a positive IGRAs result in patients with TST negative. The rate of anergic patients was also unknown because no additional skin tests to the TST were performed.

## Conclusions

Our study suggests that IGRAs, particularly T-SPOT.TB, may have a role for identifying *M. tuberculosis* infection in HIV-infected individuals, and support a dual testing approach, starting with TST and performing T-SPOT.TB if the TST is negative. Although we were unable to identify a typical patient profile with TST negative but IGRAs positive, T-SPOT.TB is more likely to be useful in patients with no past or current TB nor treatment for LTBI, a setting in which sensitivity of T-SPOT.TB was twice that of TST. Whether T-SPOT.TB truly identifies HIV-infected individuals who would benefit from preventive therapy remains unanswered. Further studies are required to establish the clinical impact and cost-effectiveness of dual testing approaches in clinical practice.

## Competing interests

The authors declare that they have no competing interests.

## Authors' contributions

Conceived and designed the experiments: JMR FG. Data collection: JMR, MM, SP. Performed the experiments: CR SB, JCR. Analyzed the data: JMR MM FG. Contributed reagents/materials/analysis tools: CR, SB, JCR. Wrote the paper: JMR MM FG. All authors discussed the results and commented on the manuscript and all read and approved the final manuscript.

## Pre-publication history

The pre-publication history for this paper can be accessed here:

http://www.biomedcentral.com/1471-2334/12/169/prepub
